# Alterations in conformational state of albumin in plasma in chronic hemodialyzed patients

**DOI:** 10.1371/journal.pone.0192268

**Published:** 2018-03-19

**Authors:** Anna Pieniazek, Lukasz Gwozdzinski, Zbigniew Zbrog, Krzysztof Gwozdzinski

**Affiliations:** 1 Department of Molecular Biophysics, Faculty of Biology and Environmental Protection, University of Lodz, Lodz, Poland; 2 Department of Pharmacology and Toxicology, Medical University of Lodz, Lodz, Poland; 3 Nephrology and Transplantation Center, Nicolas Copernic District Hospital, Łodz, Poland; University of Nebraska-Lincoln, UNITED STATES

## Abstract

**Objective:**

In chronic hemodialyzed (CH) patients the balance between production of reactive oxygen species and antioxidant defense system is disturbed and shifted towards oxidative conditions. The properties of albumin in CH patients were studied before hemodialysis (HD) and post-HD.

**Methods:**

Two oxidants were applied, organic t-butyl hydroperoxide (t-BOOH) and inorganic hydroperoxide (H_2_O_2_), for oxidation of albumin molecules. By comparison, albumin from healthy donors was also modified by both oxidants. The thiol content in albumin was determined by the Ellman method. Albumin properties were evaluated with the spin labelling technique using two covalently bound spin labels, maleimide (MSL) and iodoacetamide (ISL), and fatty acid spin probe, 16-doxylstearic acid (16-DS).

**Results:**

A decrease in thiols level in HD albumin was greater than in control albumin. The t-BOOH modified the microenvironment at the binding site of MSL and ISL in control albumin molecules to a greater extent than hydrogen peroxide. Control albumin treated with t-BOOH and H_2_O_2_ showed an increase in the mobility of 16-DS. However, no changes were observed in albumin from CH patients treated with either of the oxidizing agents.

**Conclusion:**

Both oxidants induced strong conformational changes in albumin from healthy volunteers, but were less effective or ineffective in modification of albumin derived from CH patients. These results show that albumin from CH patients is highly modified *in vivo* and is not vulnerable to oxidation in the same way as normal albumin.

## Introduction

Reactive oxygen species (ROS), including free radicals, have been suggested to contribute to many pathological conditions such as atherosclerosis, stroke, ischemia-reperfusion injury, cancer and others. Chronic hemodialysis (CH) is also associated with oxidative stress [1,2]. Oxidative stress in patients on chronic hemodialysis is manifested by an increase in lipid and protein oxidation in blood plasma, and lipid peroxidation products and protein carbonyl formation have been found in the plasma of these patients [3,4]. Hemodialyzed patients have decreased levels of antioxidant defense [5,6]. Antioxidant enzyme activities are generally lower and levels of low molecular weight antioxidants such as GSH, α-tocopherol and ascorbic acid were also lower [7]. Patients hemodialyzed using unmodified cellulose membranes develop activation of neutrophils and monocytes, which leads to respiratory burst and the release of highly reactive oxygen free radicals [8,9]. Higher levels of toxic oxygen species and impaired antioxidant defense lead to damage of blood components [2,8]. Additionally, the higher level of urea in the blood of chronic hemodialyzed patients leads to carbamylation of proteins, glycoproteins, and other components in the plasma [10].

Another negative process that may take place in patients with chronic renal failure and diabetes mellitus is glycation of proteins [4].

The physiological function of albumin is to regulate osmotic pressure and the transport of fatty acids, bilirubin, cholesterol and drugs. Albumin is the main fatty acid binding protein in plasma and possesses seven binding sites for fatty acids with moderate and high affinity [11].

It has been shown that albumin plays a key role in the antioxidative capacity of blood plasma against ROS [12,13]. Although the plasma contains ceruloplasmin and ferritin proteins that binds copper and iron ions, respectively, a high percentage of copper is bound to albumin. Metals bound to proteins are less available to participate in the Fenton and Haber-Weiss reactions. Although iron is present in plasma at much higher concentrations, copper ions reduce hydrogen peroxide 60 times faster than iron [14]. Additionally the complex of albumin with copper ions shows superoxide dismutase-like activity, preventing ROS formation [15].

In normal physiological conditions half-life of albumin is about 20 days. During this time it is permanently exposed to ROS in the blood. It has been shown that more than 70% of free radicals in plasma are trapped by this protein [16]. Albumin contains one free thiol (-SH) group (Cys-34), which accounts for 80% of the thiols in plasma. The thiol residue of Cys-34 is redox active and capable of thiolation (dithiol formation), nitrosylation and oxidation [17].

Human serum albumin is a mixture of reduced (mercaptoalbumin) and oxidized (nonmercaptoalbumin) forms [18]. Nonmercaptoalbumin (HNA) albumin consists of mixed dithiols with cysteine or glutathione and higher oxidized states (HNA ox.) contain sulfenic, sulfinic and sulfonic residues [18].

The aim of this study was to evaluate the susceptibility of plasma albumin to oxidation before, and after hemodialysis in comparison to healthy subjects. The conformational state of albumin was evaluated by electron paramagnetic resonance spectroscopy using two covalently bound spin labels, MSL and ISL and 16-doxylstearic acid as a fatty acid derivative. The level of thiols before and after oxidation in control and CH patients before and after hemodialysis were also determined. We found that normal albumin was more strongly modified by t-butyl hydroperoxide than by hydrogen peroxide. Both oxidants were less effective or ineffective in induction of structural changes in albumin derived from CH patients.

## Materials and methods

### Chemicals

4-maleimido-2,2,6,6,-tetramethylpiperidine-1-oxyl (maleimide spin label—MSL), 4-(2-iodoacetamido)-2,2,6,6-tetramethylpiperidine 1-oxyl (iodoacetamide spin label—ISL), 16-doxyl-stearic acid (16-DS), tert-butyl hydroperoxide (t-BOOH) and 5,5′-Dithiobis (2-nitrobenzoic acid) (DTNB) were obtained from Sigma-Aldrich company. All other chemicals, ammonium sulphate [(NH_4_)_2_SO_4_], sodium dihydrogen phosphate, disodium hydrogen phosphate, sodium chloride, trichloroacetic acid and hydrogen peroxide (H_2_O_2_) were purchased from POCH S.A. (Gliwice, Poland) unless otherwise indicated.

### Subjects

This survey was conducted on blood of nine hemodialyzed patients from the Department of Internal Medicine at the Medical University in Lodz. Among them eight patients had glomerulonephritis, and one had diabetic nephropathy. Patients were dialyzed using polysulfone dialyzers (LO PS 18) and dialysis fluid containing bicarbonate buffer for 3.5 to 4.5 hours, three times per week. The recruited patients (nine males) were between 39 and 74 years old, and had been undergoing hemodialysis for 57 ± 12 months (Table 1). All patients were receiving erythropoietin.

**Table 1:** Basic and biochemical data for CH patients and healthy volunteers.

	Healthy volunteers	CH patients
Parameter	mean ± SD	mean ± SD
Sex M/F	9/0	9/0
Age [years]	55 ± 5.3	58 ± 11.2
BMI [kg/m^2^]	25.9 ± 4.0	25.4 ± 3.9
Total cholesterol [mg/dl]	198 ± 39	169 ± 70
Triglycerides [mg/dl]	131 ± 77	170 ± 58
Fe [μg/dl]	89 ± 41	57 ± 28
Hb [g/dl]	13.5 ± 0.9	10.7 ± 1.7

Blood from patients was taken by their agreement and the study was approved by the Bioethical Commission of the University of Lodz (KBBN-UL/4/2012). All subjects signed an informed consent form prior to participation.

The control group of nine healthy men (45–61 years old) was recruited from among volunteers at the Outpatient Centre of Medical University in Lodz. Healthy donors used as reference group were in similar range of age as investigated patients. Chosen basic and biochemical parameters of blood of hemodialyzed patients and healthy volunteers are presented in Table 1.

Venous blood samples for experiments were collected (in standard sterile polystyrene vacuum tubes containing 16 U heparin /mL of blood) before dialysis and immediately after dialysis. Sampling of blood from CH patients and healthy volunteers took place in the morning (8:00–9:00 a.m.). Blood was centrifuged, and the plasma was collected for albumin isolation. Fasting blood samples were collected only for biochemical data of blood from CH and healthy volunteers.

### Albumin isolation

Human plasma albumin (HPA) samples were isolated, directly after blood collection (30 min) using a saturated solution of ammonium sulphate. 5 ml of the plasma was mixed on an automatic stirrer in an ice bath with slow adding drops of saturated (NH_4_)_2_SO_4_ solution. After adding 7.5 ml of ammonium sulphate, the solution was mixed for another 30 minutes. The final concentration of ammonium sulphate was 60%. Then the mixture was centrifuged at 4000 rpm for 10 min. The supernatant containing albumin was collected and dialyzed (5 ml of albumin solution to 1 L of double deionized water) using cellulose dialysis tubes membrane, MVCO 14000. Albumin solution was dialyzed twice against deionized water and a third time against PBS (5 mM, pH 7.4) (4 h each dialysis at 4 °C) on automatic stirrer to eliminate ammonium sulphate. The purification of albumin (3 mL) was continued via gel filtration using Sephadex G-75 superfine column (1.5 × 20 cm) equilibrated with PBS. The absorbance of the collected fractions of albumin was measured at 280 nm. The fractions with the highest absorbance were collected for next investigation. The protein concentration in the samples was measured (3 times in each sample) according to the method described by Lowry et al. [19].

### Modification of albumin with an oxidizing agent

The albumin isolated from healthy volunteers and CH patients was incubated with t-butyl hydroperoxide or hydrogen peroxide. Eluate obtained from column (albumin from healthy volunteers or from HD patients) was divided into three parts: control and groups treated with oxidants (t-BOOH or H_2_O_2_). Perhydrol containing 8.235 M of H_2_O_2_ was diluted with PBS to the concentration 50 mM. Then 2 μl of hydrogen peroxide (50 mM) was added to 1 ml of albumin (100 μM) (final concentration of hydrogen peroxide in albumin solution was 100 μM). Similar procedure were used for t-BOOH. Tert-butyl hydroperoxide containing 7.778 M was diluted with PBS to the concentration 50 mM. Then 2 μl of t-BOOH (50 mM) was added to 1 ml of albumin (100 μM) (final concentration of t-BOOH in albumin solution was 100 μM).

After 1h incubation albumin was dialyzed once (cellulose dialysis tubes membrane, MVCO 14000) against PBS (5 mM, pH 7.4) (4 h at 4 °C on automatic stirrer) to eliminate H_2_O_2_ and t-BOOH, respectively. At the end of dialysis the concentration of proteins were measured again (3 times in each sample) according to the method described by Lowry et al. [19]. After dialysis the thiol groups concentration (Ellman’s reaction) was measured as well as spin labeling of albumin using MSL, ISL and 16 DS was conducted.

### Thiol group concentration

The thiol group concentration in albumin was estimated using Ellman's reagent (5,5'-dithiobis-(2-nitrobenzoic acid); DTNB) [20]. The reaction product of thiol groups with Ellman's reagent is an optically active (412 nm) 2-nitro-5-thiobenzoate (NTB). Absorbance of NTB at 412 nm is directly proportional to the concentration of thiol groups in the sample.

Albumin after chemical modification was mixed with phosphate buffer (10 mM, pH 8.0) and the absorbance at 412 nm was measured (A_0_). Then the DTNB (1 M in phosphate buffer (10 mM, pH 8.0) was added to the mixture and incubated for 1 h at 37 °C in the dark. At the end of incubation the absorbance in samples were measured again at 412 nm (A_1_). The absorbance differences were calculated as follows A = A_1_ –A_0_. The calibration curve was prepared in the same way using different concentrations of reduced glutathione (GSH) as a standard instead of albumin samples. The highest concentration of GSH was 1 mM in phosphate buffer (10 mM, pH 8.0). The concentration of thiol groups was calculated based on a calibration curve and expressed as nmol/mg protein.

### Conformational state of albumin

After chemical modification the conformational state of albumin was measured using two covalently binding spin labels: MSL and ISL. In physiological conditions (pH 7.4) both reagents form covalent bonds with -SH groups, but disulfides do not react with either thiol reagent [21].

The spin label (MSL or ISL) in ethanol solution (100 mM) was added to albumin (100 μM) at the ratio 1:100 and incubated for 2 h at room temperature. The final concentration of ethanol in the samples did not exceed 0.01% (v/v). To eliminate unbound spin label, samples were dialyzed against 5 mM phosphate buffer (pH 7.4) for 24 h at 4 °C.

The h_w_/h_s_ ratio was calculated from the EPR spectra of spin labelled albumin, where h_w_—fraction of weakly immobilized spin label, h_s_—fraction of strongly immobilized spin label.

### Hydrophobic region of albumin

Changes in the hydrophobic region of albumin were estimated using a spin labelled analogue of natural stearic acid (16-doxylstearic acid). The spin label in ethanol solution (100 mM) was added to albumin (100 μM) at the ratio 0.1:100 and incubated for 30 min at room temperature. The final concentration of ethanol in the samples did not exceed 0.01% (v/v).

The h_+1_/h_0_ ratio was calculated from the EPR spectra of spin labelled albumin, where h_+1_ –height of the low-field line of spectrum, h_0_ –height of the high-field line of spectrum.

### EPR measurement

EPR spectra were performed on a Bruker ESP 300 E spectrometer at room temperature (22 ± 1 °C), operating at a microwave frequency of 9.73 GHz. The instrumental settings were as follows: centre field set at 3480 G, range 80 G, with a 100 kHz modulation frequency and modulation amplitude of 1.01 G.

### Statistical analysis

Normality of data was tested using the Shapiro-Wilk test. All data showed conformity with a normal distribution. Homogeneity of variance was verified using the Brown-Forsyth test. For variables lacking homogeneity of variance, the differences were calculated using the Kruskal-Wallis test and the data are presented as median and interquartile range (IQR: from lower quartile Q1 to upper quartile Q3). For variables with homogeneity of variance, the differences were calculated using multi-way ANOVA and post hoc Tukey test and the data are presented as mean ± standard deviation (SD) (S1 Appendix).

Statistical analysis was performed using STATISTICA.PL v.12.5 (StatSoft POLSKA).

## Results

**Fig 1:**
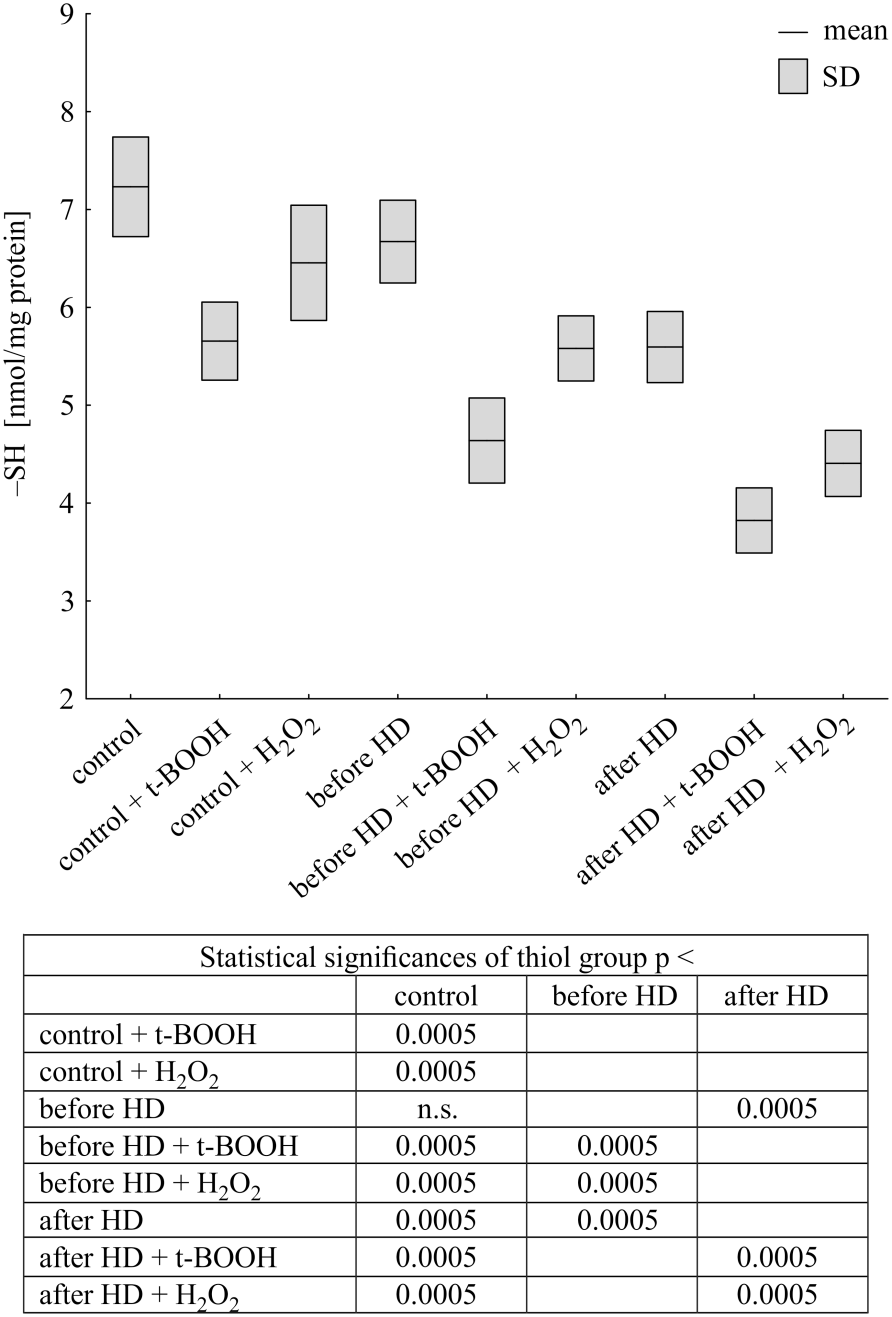
Changes in thiol group levels of albumin isolated from CH patients and healthy volunteers, treated with t-butyl hydroperoxide and hydrogen peroxide. Data expressed as mean ± SD.

In our study, applied concentrations of hydrogen peroxide or t-BOOH did not oxidize all thiol groups in albumin. The thiol content in albumin samples was determined by spectrophotometric measurements using Ellman’s method. The obtained data showed the effect of hemodialysis on the level of –SH groups in CH patients. After HD the level of thiols was significantly decreased (approx. 15%). As expected, both oxidants led to a significant decrease in the thiol groups in albumin. However, a significantly greater decrease in thiol content was observed following t-BOOH treatment than H_2_O_2_ treatment, at 23% and 10% respectively in control (normal) albumin (Fig 1). In albumin from CH patients an even higher decrease was found, 33% upon t-BOOH treatment and 18% upon H_2_O_2_ treatment before hemodialysis, and 31% and 24%, respectively after hemodialysis.

**Fig 2:**
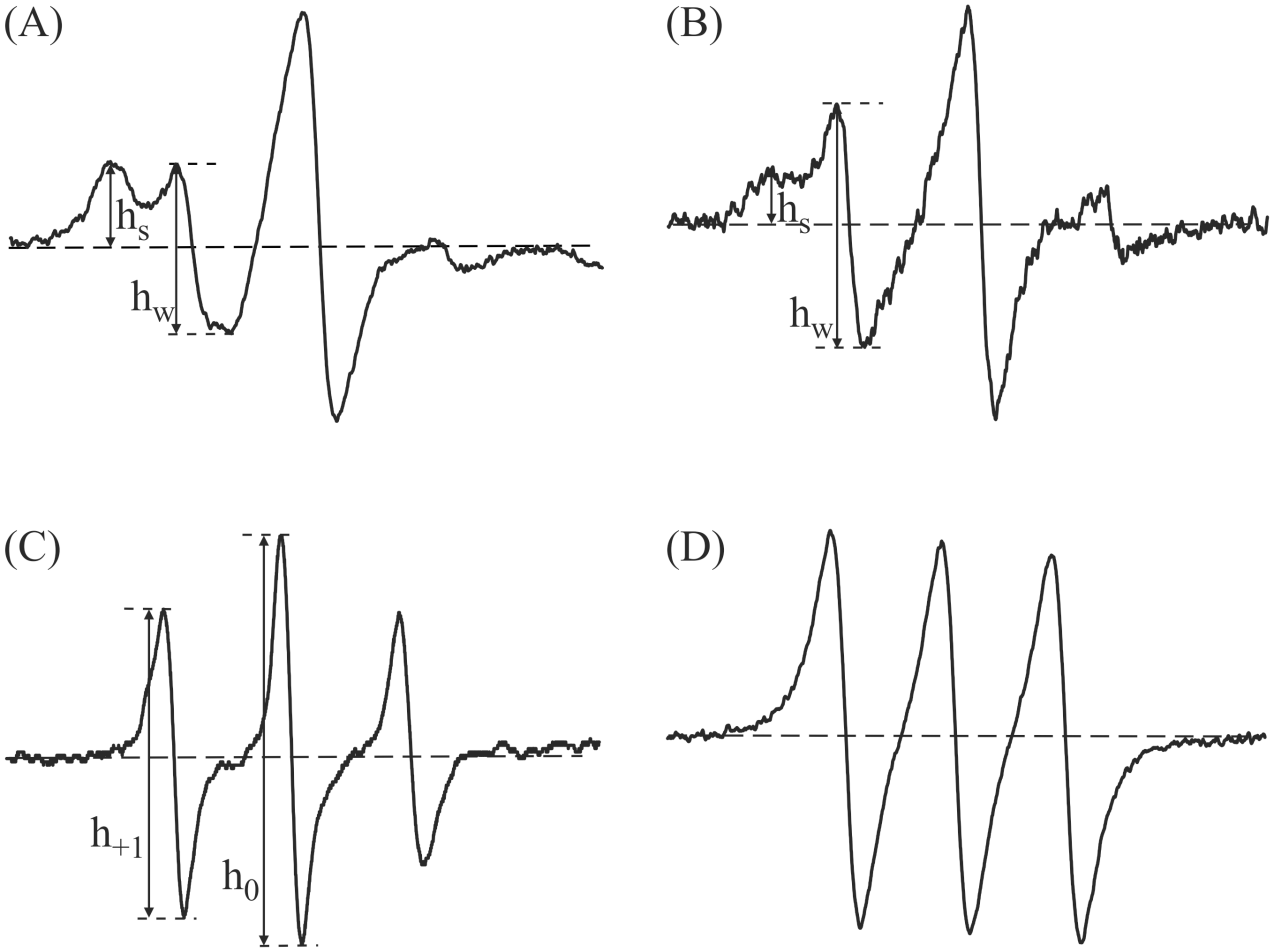
EPR spectra of MSL (A); ISL (B); 16 doxyl stearic acid (C) attached to albumin and 16- doxylstearic acid dissolved in heptane (D).

Although oxidation led to a decrease in thiol groups, both spin labels MSL and ISL were attached to albumin molecules (Fig 2A and 2B).

The EPR spectra of spin-labeled albumin with MSL and ISL showed that the spin labels are attached to at least two different binding sites. Complex of low-field line of EPR spectra exhibited wide band of strongly immobilized component (h_s_) and narrow line (h_w_), weakly immobilized component (Fig 2A and 2B). There was a major signal, A, which characterized a strongly immobilized environment and a minor signal, B, which characterized a weakly immobilized environment.

Information on the conformational state of proteins can be obtained from the ratio of the ESR spectral amplitude of the low field resonance line of spin labelled albumin (MSL and ISL) attached to weakly immobilized binding sites (h_w_) to that attached to strongly immobilized binding sites (h_s_). This ratio is highly sensitive to many experimental parameters, such as pH, influence of various ligands, the temperature and other factors in the EPR measurements [22]. However, the EPR spectra showed differences in immobilization of both labels in the microenvironment of the binding site. Use of MSL and ISL reflected changes in albumin structure upon both oxidizing agents treatment.

**Fig 3:**
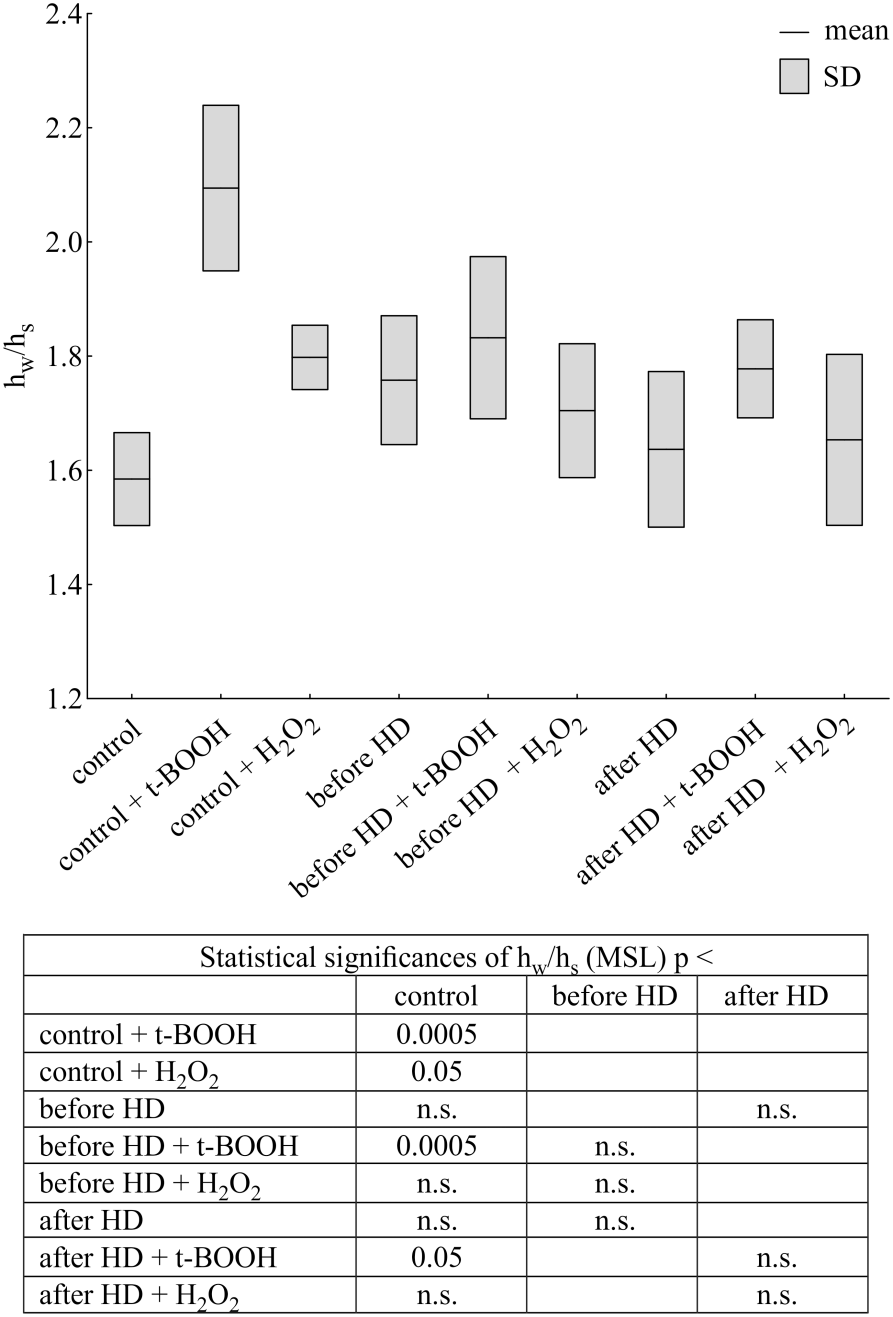
Changes in h_w_/h_s_ ratio of maleimide spin label attached to albumin isolated from CH patients and healthy volunteers treated with t-butyl hydroperoxide or hydrogen peroxide. Data expressed as mean ± SD.

In the control albumin h_w_/h_s_ ratio increased after t-butyl hydroperoxide (31% vs. control) treatment (Fig 3). This organic peroxide modified the microenvironment at the binding site of MSL in albumin molecules to a greater extent than hydrogen peroxide (13% vs. control). On the other hand, there was a slight, non-significant increase in this parameter in albumin from CH patients upon t-BOOH treatment before and after hemodialysis in comparison to HD patients, but a significant increase vs. albumin from healthy donors (Fig 3).

**Fig 4:**
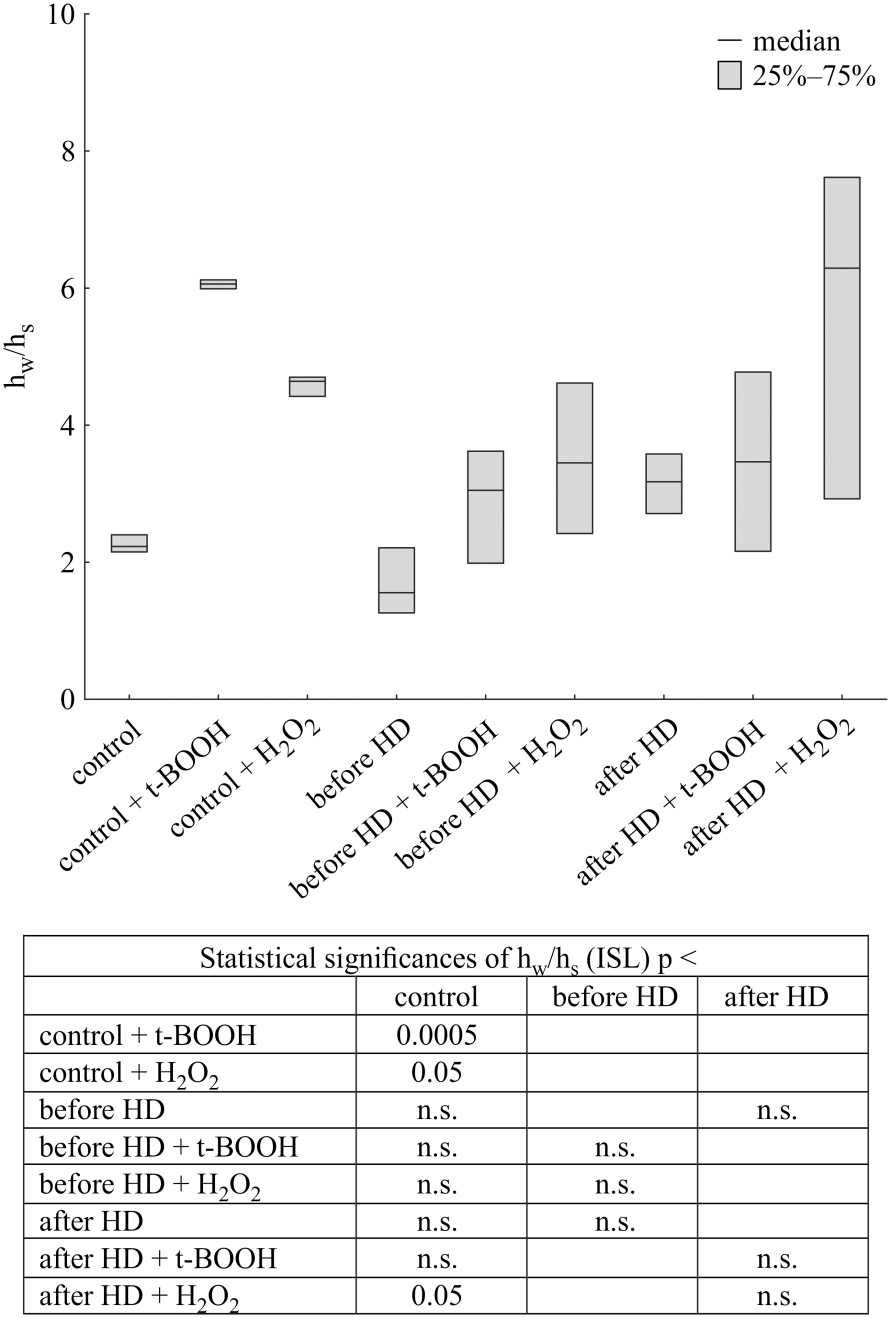
Changes in h_w_/h_s_ ratio of iodoacetamide spin label attached to albumin isolated from CH patients and healthy volunteers treated with tert-butyl hydroperoxide or hydrogen peroxide. Data presented as median and interquartile range.

In the case of ISL there was an approx. 3-fold and 2-fold increase in the h_w_/h_s_ ratio after t-BOOH and H_2_O_2_ treatment of native albumin, respectively (Fig 4). However, there was no difference in albumin from CH patients after treatment with either oxidant before hemodialysis but a significant increase (in comparison to control) in this parameter after hemodialysis upon H_2_O_2_ treatment (Fig 4).

Spin labelled fatty acid attached to albumin showed a triplet spectrum (Fig 2C). Fig 2D illustrates the rotation of this label bound to this protein and free rotation of the label in heptane. For the estimation of the motion of 16-DS attached to albumin the h_+ 1_/h_0_ ratio was calculated [23]. The EPR spectrum of albumin labeled with 16-DS clearly shows the binding of this label with HPA without the presence of free label rotation in both native and hemodialyzed patients albumin. When the ratio of fatty acids to albumin increases, the EPR spectrum reveals freely tumbling component.

**Fig 5:**
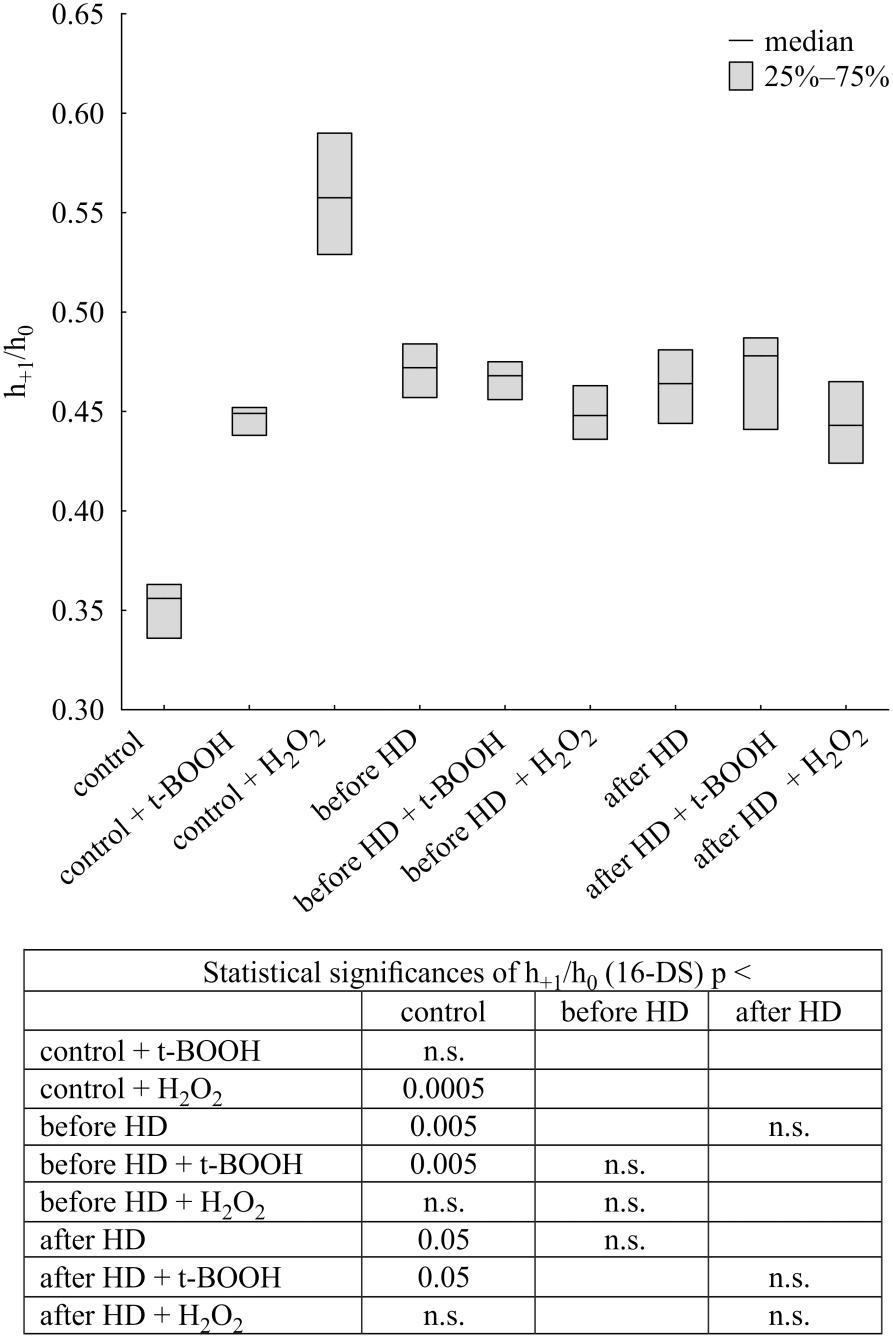
Changes in h_+1_/h_0_ ratio of 16-doxylstearic acid bound to albumin isolated from CH patients and healthy volunteers treated with t-butyl hydroperoxide or hydrogen peroxide. Data presented as median and interquartile range.

Large changes in the control albumin structure were observed after treatment with both oxidants, using a 16-DS spin probe. Control albumin treated with t-BOOH showed an approx. 1.3-fold increase in the h_+1_/h_0_ ratio and an approx. 1.6-fold upon hydrogen peroxide treatment. On the other hand, there were no changes in albumin from CH patients treated with either oxidizing agent. In albumin from HD patients there was a significant increase in the h_+1_/h_0_ ratio before and after hemodialysis as well as upon t-BOOH treatment only vs. the control (Fig 5).

## Discussion

Albumin plays a key role in the regulation of osmotic pressure, and the binding and transport of various substances including bilirubin, steroids, fatty acids and drugs. The Cys-34 in HPA is the most abundant plasma thiol which has central contribution of albumin redox processes [24]. This protein also plays an important role as a scavenger of reactive oxygen species and as a transition metal binding protein, mainly copper [12]. Albumin may be oxidatively modified *in vivo* in chronic kidney disease (hemodialyzed patients), diabetes mellitus liver disease and other diseases.

In this paper we investigated the properties of albumin after oxidation from healthy and hemodialyzed patients before and after hemodialysis. Albumin from both sources was modified by oxidation using t-butyl hydroperoxide and hydrogen peroxide.

It has been shown that hydrogen peroxide reacts with HPA thiol at pH 7.4 stoichiometrically (1:1) forming sulfenic acid derivative without intermolecular disulfide dimers [25]. On the other hand the formation of albumin disulfide dimers following t-butyl hydroperoxide exposure was also observed [26]. The hydrogen peroxide or t-butyl hydroperoxide concentrations used in these studies did not oxidize all thiols in albumin. We estimated that 100 μM of both reagents oxidized approx. 55% of thiols. It was reported that 300 μM of H_2_O_2_ oxidized all thiols in albumin [25].

The level of thiol groups in control albumin reveals its decrease after t-BOOH and H_2_O_2_ treatment. The decrease in thiols level in albumin after hemodialysis compared to before hemodialysis was observed. Higher oxidation of albumin after hemodialysis may be connected with decrease of antioxidant capacity of plasma, what was showed by Ruskovska and colleagues [27]. The addition of both oxidants to CH albumin leads to further diminishing of thiol groups in albumin before and after hemodialysis. Tert-butyl hydroperoxide induced higher decrease of –SH groups than hydrogen peroxide in albumin from healthy and CH donors. The greater effectiveness of t-BOOH in the oxidation of thiol groups is related to its greater hydrophobicity compared to hydrogen peroxide. T-BOOH penetrates more easily into the Cys-34 residue in albumin, which is located in 10 angstrom wide hydrophobic pocket [28]. It seems that pre-oxidation (*in vivo*) of albumin in HD patients causes increased sensitivity for its oxidation. Especially this process is visible after hemodialysis.

Using the spin labelling method we investigated the environment at the binding site of Cys34 as well as hydrophobic regions in this protein, which bind fatty acids. Two spin labels, maleimide and iodoacetamide, form covalent bonds with the –SH group of Cys-34, while binding of 16-doxylstearic acid was associated with hydrophobic regions of albumin molecules, similarly to natural saturated fatty acids, which are transported by this protein. Depending on the level of oxidation state of albumin, binding of fatty acids or drugs can be decreased [29].

Although both spin labels, MSL and ISL react with Cys34, the EPR spectra showed differences in their immobilization in the microenvironment of the binding site. The spectrum of maleimide attached to albumin exhibited a higher degree of immobilization than that of iodoacetamide. In the ISL spectrum, the participation of a strongly immobilized component is smaller than in the MSL spectrum.

A drop in the level of thiol groups was accompanied by changes in the conformational state of the albumin upon both oxidants treatment. Deeper alterations were found in normal albumin than those from HD patients, indicated by maleimide spin label. More extensive changes in control albumin structure after oxidation were observed when using iodoacetamide derivative of nitroxide. Both oxidants has also influenced on CH albumin.

Generally, we found that t-BOOH was more effective at damaging the region containing the cysteine residue in normal albumin molecules than hydrogen peroxide. Different action of both oxidants was observed in peroxiredoxin (Prx-4) treatment. T-BOOH completely inhibited Prx-4, while H_2_O_2_ treatment maintained the peroxidase activity [30]. On the other hand, both oxidants resulted in the same oxidative damage leading to sulfinic acid formation in cysteine residue [30].

It has been shown that 16-doxylstearic acid was used for the study of conformational changes of albumin and it is highly specific for HPA [31,32]. Crystallographic analysis showed that human serum albumin possesses seven medium-chain and long-chain (C10–C18) saturated fatty acid and unsaturated fatty acid binding sites [33,34]. Additionally, NMR studies revealed high-affinity binding pockets for fatty acids on the protein [35]. It has been shown that in physiological condition albumin binds 0.1–2 mol of fatty acid per mol of protein [36].

Strong alterations in conformational state of normal albumin after oxidation were also observed using 16-doxylstearic acid label, but all changes in the HD albumin structure were not statistically significant either before or after hemodialysis. However, the fatty acids-binding regions was more strongly modified by hydrogen peroxide than t-BOOH, as demonstrated by 16-DS studies.

Analysis of EPR spectra of MSL and ISL revealed an increase in the mobility of both spin labels attached to albumin after peroxide treatment. Similar results were obtained for 16-DS in hydrophobic regions binding fatty acids. These results reflected alterations in two regions of the bound spin labels and showed conformational changes in the albumin molecule after oxidation associated with loosening of its structure. The first concerns the microenvironment of Cys-34. The second is associated with three main sites in the albumin molecule responsible for binding long-chain fatty acids with high affinity, containing Lys 351, Lys 475 and Arg 117 (domain IA, IB, IIB) [12,33,36]. However, it has been shown that 16-doxylstearic acid is bound to the domain IIB of albumin molecule [32]. Our results are in line with Kawakami and colleagues [29] who observed conformational changes in oxidized albumin in comparison to normal protein.

The redox state of albumin have a critical influence on ligand binding properties including drugs, fatty acids and bilirubin [37]. Oxidized albumin indicates also lower antioxidant properties in free radicals scavenging activities and may impair albumin function in a number of pathological conditions [29]. I has been reported that HD patients oxidized albumin might enhance oxidative stress [38]. However, in the case of chronically hemodialyzed patients, albumin can be damaged *in vivo* not only by hydrogen peroxide or organic hydroperoxide but also other oxidative species. During hemodialysis activation of neutrophils and monocytes by dialyzed membranes leads to respiratory burst and release of reactive oxygen species such as superoxide anion radical (O_2_**˙**^–^), hydroxyl (HO**˙**) radical, nitric oxide (NO**˙**), hydrogen peroxide, and hypochlorous acid (HClO). Using EPR spectroscopy we demonstrated superoxide and hydroxyl radical release during hemodialysis [8,9]. In these conditions ROS can oxidize/damage biological materials including albumin. It has been shown that albumin scavenges hydroxyl radicals [12]. Oxidation of this protein leads to the formation of dithiols, and stronger oxidation to sulfenic, sulfinic and sulfonic acids [37,39]. In our previous work we showed a decrease in thiols and an increase in hydroperoxide and protein carbonyl levels as well as glycosylated proteins in the plasma of hemodialyzed patients [4]. Generation of superoxide and nitric oxide leads to the formation of peroxynitrite, another powerful oxidant and nitrating agent [40]. Eiserich and colleagues showed another pathway of nitration by neutrophils, which promote nitration by converting nitrite into nitryl chloride (NO_2_Cl) and nitrogen dioxide (NO_2_), both nitrating and oxidizing agents, through myeloperoxidase-dependent pathways [41]. In condition described above plasma proteins of CH patients are post-translationally modified by nitration of their tyrosine residues [42]. The thiol group of albumin plays an important role as an antioxidant against peroxynitrite and was shown to be oxidized to a sulfenic acid (HSA-SOH) [25]. Nitric oxide (NO) may be overproduced in hemodialyzed patients [43]. NO can react with thiols to produce nitroso-derivatives. Nitroso compounds including nitroso-albumin were found in human plasma and whole blood [44,45].

Another strong oxidizing agent is HClO, released by neutrophils during oxidation of chlorides in the presence of myeloperoxidase [46]. It has been shown that albumin is able to scavenge this species [46]. In addition, hypochlorous acid leads to modification of aromatic amino acids such as tryptophan and tyrosine [47,48]. It has been reported that the increase in protein carbonyls in the plasma of hemodialyzed patients was correlated with an increase in oxidized albumin [38]. Relative to other plasma proteins and lipoproteins, albumin was much more oxidized in CH than in healthy volunteers [49,50]. The authors suggested that albumin is the major plasma protein target of oxidant stress in CH patients.

Mechanical forces during hemodialysis generated by pumps disrupt erythrocytes and release hemoglobin, which is a powerful catalyst [51,52]. Autoxidation of oxyhemoglobin [Hb(Fe^2+^)O_2_] to methemoglobin (MetHbFe^3+^) generates superoxide anion radicals, which dismutate to hydrogen peroxide. Oxidation of both Hb(Fe^2+^)O_2_ and (MetHbFe^3+^) by H_2_O_2_ leads to highly reactive intermediates, the oxoferryl complex HbFe^4+^ = O and HbFe^4+^ = O^.^(ferryl radical form), respectively [53,54]. Ferryl Hb and ferryl radicals can oxidize biological materials *in vivo* and these reactions have been implicated in diverse pathophysiologies [55]. It seems that in CH patients oxidative stress induced by this pathway is important in plasma protein damage, including that of albumin.

HPLC analysis revealed two oxidized fractions of albumin from HD patients, one of these consisting mainly of mixed dithiols with cysteine and glutathione, while the second fraction consisted of more oxidized thiols such as sulfenic, sulfinic and sulfonic acid residues [18].

## Conclusion

In this work we showed strong impact of both oxidants on conformational state of albumin only from healthy volunteers. These results showed that albumin from hemodialyzed patients is highly modified *in vivo* and is not vulnerable to oxidation as albumin from healthy volunteers *in vitro*. The different mechanism of action of both oxidants may be related to their different hydrophobicity.

## Supporting information

S1 Appendix Dataset (Excel). (XLS)
